# The Future of Metformin in the Prevention of Diabetes-Related Osteoporosis

**DOI:** 10.7759/cureus.10412

**Published:** 2020-09-12

**Authors:** Myat Aung, Saba Amin, Azouba Gulraiz, Fenil R Gandhi, Julio A Pena Escobar, Bilal Haider Malik

**Affiliations:** 1 Medicine, California Institute of Behavioral Neurosciences & Psychology, Fairfield, USA; 2 Emergency Department, Poole Hospital, Poole, GBR

**Keywords:** metformin, osteoporosis, ampk, diabetes, bone disorder, osteogenesis

## Abstract

As a worldwide aging population is on the rise, osteoporosis (OS) is becoming a global health burden. Therefore, many researchers and health authorities are looking into the potential prevention and treatment of OS. Although previously regarded as two separate pathological processes, diabetes (DM) and OS are now regarded as two conditions that can occur together. It is now believed that OS can develop as a complication of DM. This relationship is further evidenced through a reduction in bone mineral density in type-1 diabetes with a resulting increased risk of fracture. Although bone mineral density in type-2 diabetes mellitus is normal or increased, there is also increased fragility due to decreased bone quality. These abnormal bone qualities tend to occur through the production of reduced bone microvasculature and advanced glycation end product, AGE. Interestingly, one of the most common treatments for DM, metformin (MF), shows a promising result on the protection of diabetes and non-diabetes related bone turnover. It is believed that MF modulates its effect through the adenosine monophosphate-activated protein kinase (AMPK) pathway. Recent data regarded AMPK as a vital mediator of homeostasis. It is involved not only in glucose metabolism but also in osteogenesis. AMPK can directly influence the production of mature and good quality bone by decreasing osteoclasts, increasing osteoblast formation, and enhancing bone mineral deposition. As an activator of AMPK, MF also upregulates osteogenesis. Furthermore, MF can influence osteogenesis through a non-AMPK pathway, such as the fructose 1-6 phosphatase pathway, by reducing glucose levels. While already recognized as a safe and effective treatment for DM, this article discusses whether MF can be used for the prevention and treatment of OS.

## Introduction and background

Osteoporosis (OS) is the pathology of bone characterized by a decrease in the overall quality of the bone, which can eventually lead to an increased risk of fracture [1]. The prevalence of OS tends to increase with age. Adults over the age of 50, one-third of the female population and one-fifth of the male population is affected by an osteoporosis-related fracture [2]. It is estimated that approximately 125 million people living in Europe, India, Japan, and the USA have OS [2]. Similarly, diabetes mellitus (DM) is a global health burden affecting nearly 422 million individuals around the planet [3]. Also, the chances of acquiring DM increases with age.

Many recent published papers have started to recognize the relationship between OS and DM. Bone mineral density (BMD) in type-1 diabetes mellitus (T1DM) is decreased, subsequently leading to increased bone fragility [4-5]. BMD in type-2 DM (T2DM) is either normal [6] or elevated [5]. However, the prevalence of fractures in T2DM is also high, which might be due to multiple confounding mechanisms such as anti-diabetic medications, associated renal pathology, genetics, and hypo- or hyperglycaemic-related fall [7]. The risk of fracture in T2DM is notably higher in elderly menopausal women [8-9].

The principle treatment for T1DM, insulin, has been shown to increase the occurrence of bone fracture [8]. Many studies suggest that thiazolidinediones (TZD), such as rosiglitazone and pioglitazone, enhance osteoporotic-related fragility and hence should be avoided [8,10]. A newer oral hypoglycaemic agent, sodium-glucose transport protein 2 inhibitors, SGLT2 inhibitors, which include canagliflozin, dapagliflozin, and empagliflozin, should also be used with caution because of its unclear tendency toward fragility [8,10]. However, the most commonly used biguanide class of anti-diabetic drug, metformin (MF), has shown potential in preventing osteoporosis-related fractures [11].

MF modulates bone quality through many receptors, including adenosine monophosphate-activated protein kinase (AMPK) [12], which is of utmost importance for bone regeneration [11]. MF also inhibits advanced glycation end product (AGE), which can promote bone turnover [13]. A recent study even suggests that MF is beneficial for bone health in adult women without T2DM and obesity [14]. Since its discovery, MF has shown desirable lipid-lowering activity [10] and cardio-protective effect [15]. Its role in oncology has been steadily increasing over the last few decades because an increased overall survival rate is observed in various malignancies such as pancreatic, colorectal, breast, liver, and lung cancer [16]. In this article, we will emphasize mainly on the anti-osteoporotic effect of MF and discuss whether this biguanide medication can be used as prophylaxis for osteoporosis.

Methods

The search terms included in this review article are “metformin”, “osteoporosis”, “Diabetes”, “type 2 diabetes”, “type 1 diabetes”, “Fracture”, “hepatic gluconeogenesis”, “AMPK”, “glucose-lowering agent”, “advanced glycation product”, “bone metabolism”, “osteogenesis”, “osteoblast”, “osteoclast”, “RANKL”, “PPAR,” and “Fructose 1-6 phosphatase.” The PubMed, Cochrane, Google Scholar, and University of Manchester online libraries were used to search for articles. Studies included in this paper are review articles, RCTs, systematic reviews, and meta-analyses.

## Review

Osteoporotic fractures and diabetes

It has been many years since clinical researchers proved the link between DM and OS [5,8,17]. More recently, researchers are emphasizing the biochemical effect of DM on OS. An animal study suggests that diabetic condition deteriorates bone health by disturbing the trabecular bone architecture and BMD [18]. In an individual with T1DM, reduction in BMD can be observed and, therefore, it is reasonable that T1DM can lead to an increased risk of fracture [4-5]. However, in T2DM, normal or increased BMD can be observed [5-6]. Despite this fact, evidence suggests that the risk of fracture is higher in T2DM as compared to a non-diabetic patient [9,17-18]. These facts prove that more complex biochemical activities are causing the fragility, particularly in T2DM.

Hyperglycemia induces the production of reactive oxygen species (ROS) and AGE [19]. These products can cause oxidative stress leading to abnormal bone homeostasis [20]. Osteoblast, the bone-forming cells were inhibited and osteoclast stimulated by AGEs, causing increased bone turnover [21]. Osteoblasts derive from mesenchymal stem cells (MSC), which is the common ancestor of adipocytes. Osteoclasts develop from hematopoietic stem cells (HSC) [22]. With impaired glucose metabolism, an essential nuclear receptor called peroxisome proliferative activated receptor (PPARγ) [10] causes an increased bone turnover by diverting MSC towards adipocytes and increases proliferation of HSC [23-24].

The bone was conventionally regarded as an organ to support the body and to protect the internal organs. More recently, researchers appreciated the endocrine activities of the skeleton [25]. Osteocalcin is a protein produced by mature osteoblast and is regarded as a marker of bone formation [25]. This protein plays an important role in glucose metabolism by enhancing insulin secretion by pancreatic B cells and increasing insulin sensitivity [25-26]. Therefore, not only does diabetes play a role in the development of osteoporosis, but bone also regulates the development of diabetes. Some complications of diabetes could indirectly lead to osteoporotic fracture. One animal studies suggest that diabetes disrupt the trabecular architecture of femoral head, therefore leading to higher chance osteoporotic fragility in diabetic rat than control [18]. Diabetes can also induce osteoporotic fragility through diabetic nephropathy [7] and non-alcoholic liver disease [27]. Also, lower limb muscle strength is much weaker in T2DM patients with osteoporosis than patients with T2DM alone [28]. One has also to consider that the fall initiated by the hypoglycaemic attack can also lead to osteoporotic fracture, especially in elderly postmenopausal T2DM women [8-9].

Another important mechanism that needs a detailed discussion is the AMPK pathway, whose relation with diabetes and osteoporosis will be later explained in the following section of this article. In short, AMPK is a signaling pathway that is an essential regulator of homeostasis and regarded as a potential target for the treatment of endocrine or metabolic disease, including DM [29]. This critical pathway, once activated, was shown to decrease osteoporotic related bone loss in ovariectomized rats [30]. This process seems to occur through the receptor activator of nuclear factor-kappa-Β ligand (RANKL)-related increased osteoclast formation, which is inhibited by the AMPK pathway [30]. Many drugs can activate the AMPK pathway and, interestingly, MF is among these medications [31].

Overview of the AMPK pathway

Many researchers regarded AMPK as an energy sensor. AMPK contains three subunits (α, β, γ), which can lead to many forms of isomers [29]. There are two α, two β, and three γ subunits [29]. The pathway is believed to be initiated by the binding of adenosine monophosphate (AMP) to one of these subunits mainly to γ subunits, and also through phosphorylation, mainly of α subunit [32]. Once bound and activated, AMPK generates energy in the form of adenosine triphosphate (ATP) [29,33]. Therefore, the AMPK pathway depends on the AMP/ATP ratio: an increase in the AMP/ATP ratio will lead to activation while a decrease in the ratio will lead to the inhibition of the pathway [29,32-33].

Although the AMPK pathway act as a regulator of metabolism, there are a few systems that control AMPK. Liver kinase B1 (LKB1) is a protein kinase that has a regulatory effect on AMPK [32]. It is also believed that MF has regulatory properties on AMPK through LKB1 [32]. Another pathway that has an effect of AMPK is calmodulin-dependent protein kinase kinase (CaMKKbeta), which activates via similar phosphorylation activity [33-34]. A more recent finding suggests some molecules can directly activate AMPK such as a small molecule numbered as A-769662 and an enzyme called 5-aminoimidazole-4-carboxamide-1-β-d-ribofuranoside (AICAR) [35]. Also, fructose-1, 6-bisphosphate can have a positive mediation effect on AMPK, which can lower liver glucose production [36].

AMPK and osteogenesis

As a pathway for stabilizing homeostasis, AMPK is involved in osteogenesis. Lipid-forming adipocytes and bone-forming osteoblasts have a common ancestor, mesenchymal stromal cells (MSC) [22]. Depending on the body’s signal, MSC can either differentiate into adipocytes or osteoblasts. The stimulation of AMPK can lead to the differentiation of MSC into osteoblasts, thereby increasing bone formation [12]. AMPK induces these effects through the runt-related transcription factor Runx2 [37], which is vital for the maturation of chondrocyte and healthy skeletal formation [22]. Also, the inhibition of AMPK led to a decrease in the production of alkaline phosphatase, osteocalcin, and type-1 collagen, all of which are important for good quality bone formation [38]. It is also shown that AMPK inhibition would lead to decreased cellular activity of alkaline phosphatase and Runx2 [38].

Another important regulator of osteogenesis that correlates with AMPK is peroxisome proliferator-activated receptors (PPAR). PPAR can direct MSC toward adipocytes, thereby reducing bone formation [24]. Interestingly, a few recent studies have shown that the activation of AMPK by pharmacological means can lead to decreased activity of PPAR, ultimately leading the MSC cell line toward osteoblast formation. [22,24,37].

However, bone homeostasis involves not only osteoblasts but also osteoclasts. In contrast to osteoblasts, osteoclasts are bone-absorbing cells. These cells derive from hemopoietic precursor cells. An essential regulator of an osteoclast cell line is the receptor activator of the nuclear factor-kappa-Β ligand (RANKL) pathway [39]. Recent studies suggest that AMPK decreases osteoclast formation by means of autophagy and the downregulation of the RANKL pathway [40-41].

As shown in Table 1, the activation of AMPK ultimately leads to increased catabolism by increasing cellular glucose uptake, fatty acid oxidation, and decreased glycogen synthesis while decreasing anabolism by reducing fatty acid, protein, and cholesterol synthesis [11,29,33]. Any activity that requires energy will tend to activate the AMPK pathway. Therefore, exercise can initiate the AMPK pathway [11].

**Table 1:** AMPK pathway The initiation of AMPK will lead to an increase in cellular catabolism and a decrease in anabolism. AMPK=AMP activated protein kinase; AMP=adenosine monophosphate

AMPK pathway	
Increase catabolism	Decrease anabolism
Increase cellular glucose uptake	Decreased fatty acid synthesis
Increased fatty acid oxidation	Decreased protein synthesis
Decreased cellular storage of glycogen	Decreased cholesterol synthesis
Decreased synthesis of glycogen	

As a regulator of metabolic activity, many researchers started to appreciate the role of AMPK and tried to target this pathway as a treatment of many metabolic diseases such as DM and OS. Many substances have been shown to have activity on AMPK [16,20,38,42]. Among them, MF, which is already being used as a first-line medication for DM, shows a positive regulation effect of AMPK [37,42-43].

Metformin (past, present, and future)

Metformin was created in 1922. MF has a strong relationship with guanidine, whose concentration is abundantly found in Galega Officinalis, also known in Europe as goat’s rue [44]. It is included in a biguanide group of medication (1-1 dimethyl-biguanide) [44]. After its discovery, it has shown glucose-lowering activity. Then, a French scientist, Jean Sterne, started the clinical trial for the treatment of diabetes during 1950 [44]. After showing promising results, it was authorized in the US by the 1990s for the treatment of diabetes [44]. MF is one of the most commonly utilized medications in the world. MF is also included in the WHO list of essential medicines [45]. The reasons for its widespread use is cost-effectiveness, safety profile, and the easy method of delivery (orally) [10]. MF also has superiority over other anti-DM medications, such as sulphonylurea, thiazolidinediones, dipeptidyl peptidase 4 (DPP-4) inhibitors, and alpha-glucosidase inhibitors, in term of less hypoglycaemic risk and weight gain [46]. It is the first-line drug for T2DM, either used alone or in combination with the above-mentioned anti-diabetic medication. However, despite its widespread use, the mechanism of action of this century-long medication was not fully understood yet.

Relationship between metformin and the AMPK pathway

Recent advances in biomedical technology allow researchers to gain further insight into the detailed mechanism of MF. Some recent studies suggest that MF exerts its effect through AMPK and non-AMPK-related pathways [47-48].

Many data indicated that MF exerts its anti-diabetic effect through the activation of AMPK [42-43]. MF once entered the bloodstream, inhibits complex-1 in mitochondrial, thereby reducing the synthesis of ATP by mitochondria [48-49]. This process increases the ADP:ATP (adenosine diphosphate:adenosine triphosphate) ratio inside the cytoplasm [29,32,48-49]. As mentioned above, an increase in this ratio can activate the AMPK pathway. Once activated, this will lead to increased cellular catabolism and decreased anabolism [48].

Effect of metformin on osteogenesis (AMPK-dependent pathway)

The skeleton protects internal organs and provides support to the human body. Bone formation and breakdown is regulated by osteoclasts and osteoblasts. The overproduction or under secretion of these cells will lead to one or some of several life-changing pathologies such as cancer, osteoporosis, and osteopenia. Therefore, bone homeostasis is of utter importance.

MF is believed to involve in osteogenesis by utilizing the AMPK pathways [37]. Many articles shown that MF is a potent stimulator of AMPK [11-12,15,29,31,34,42-43]. The suppression of ATP production by inhibiting complex-1 in mitochondria can increase the ADP:ATP ratio [48-49]. This activity will commence the AMPK pathway. Then, AMPK will influence bone formation by directing MSC toward osteoblast production rather than adipocyte [37]. Another important consequence of this activation is the phosphorylation of acetyl coenzyme A (Acetyl-CoA1) by AMPK [47]. This process is related to the cholesterol-lowering and the insulin-sensitization effect of MF [47]. Along the cascade of AMPK activation, MF can influence its osteogenesis activity through Runx2 [37]. This will cause mature osteocyte formation and proper bone mineralization [38]

Some studies prove these osteogenic effects. MF is shown to decrease bone turnover when compared with other anti-diabetic medications [48]. Also, the deterioration of bone microarchitecture caused by insulin deficiency can be prevented by the intake of MF [49].

AMPK independent pathways

DM can deteriorate bone quality by causing a disturbance in intracellular glucose metabolism. Bad bone quality in DM can be explained by decreased insulin activity and hyperglycemia, which can cause reduced skeletal microvasculature formation and deposition of AGE in bone [10,13]. The impairment of osteoblastic function by AGE can increase the risk of fracture in a diabetic patient [13].

By decreasing hepatic gluconeogenesis and by increasing insulin sensitivity and secretion, MF can improve bone quality by reducing the glucose level in the body. This process is thought to be independent of AMPK and believed, so far, to involve the inhibition of fructose 1,6-biphosphatase in hepatocyte [37,50].

## Conclusions

Much research proves that DM can increase the prevalence of OS. However, this article demonstrates that one of the primary treatments of DM - MF - can interestingly decrease the occurrence of OS. Although the mechanism of action was previously unknown, with recent advances in medical and pharmacological technology, it is now proven that MF has a stimulatory effect on the AMPK pathway. Through this pathway, MF influences the bone homeostasis benefits. At the same time, this article shows that AMPK is involved not only in osteogenesis but also in the treatment of DM, and several modulatory mechanisms are being developed to target this essential pathway. Moreover, the AMPK-independent behavior of MF is discussed in this article; MF reduces poor quality bone formation as a result of abnormal glucose levels. Fructose 1,6-biphosphatase is also an important pathway found in hepatocytes, controlled by MF, increasing insulin activity in the body and ultimately leading to optimum osteogenesis. This article provides grounds for further research in the prevention of the global health burden and OS, and on whether MF is the future medication in the prevention of this pathology. Additional research is fundamental in the future to determine whether MF can be used either alone or in combination with other anti-osteoporosis drugs. Also, the dosage of MF is to be focused on its therapeutic effect. Ultimately, well-designed, double-blinded, randomized controlled trials have to be carried out to prove the efficacy and safety of MF for the prevention and treatment of osteoporosis and osteoporosis-related fractures.
